# Patterning a Leaf by Establishing Polarities

**DOI:** 10.3389/fpls.2020.568730

**Published:** 2020-10-30

**Authors:** Darren Manuela, Mingli Xu

**Affiliations:** Department of Biological Sciences, University of South Carolina, Columbia, SC, United States

**Keywords:** adaxial–abaxial polarity, proximodistal polarity, mediolateral polarity, leaf, lamina outgrowth

## Abstract

Leaves are the major organ for photosynthesis in most land plants, and leaf structure is optimized for the maximum capture of sunlight and gas exchange. Three polarity axes, the adaxial–abaxial axis, the proximal-distal axis, and the medial-lateral axis are established during leaf development to give rise to a flattened lamina with a large area for photosynthesis and blades that are extended on petioles for maximum sunlight. Adaxial cells are elongated, tightly packed cells with many chloroplasts, and their fate is specified by HD-ZIP III and related factors. Abaxial cells are rounder and loosely packed cells and their fate is established and maintained by YABBY family and KANADI family proteins. The activities of adaxial and abaxial regulators are coordinated by ASYMMETRIC LEAVES2 and auxin. Establishment of the proximodistal axis involves the BTB/POZ domain proteins BLADE-ON-PETIOLE1 and 2, whereas homeobox genes *PRESSED FLOWER* and *WUSCHEL-RELATED HOMEOBOX1* mediate leaf development along the mediolateral axis. This review summarizes recent advances in leaf polarity establishment with a focus on the regulatory networks involved.

## Introduction

**FIGURE 1:**
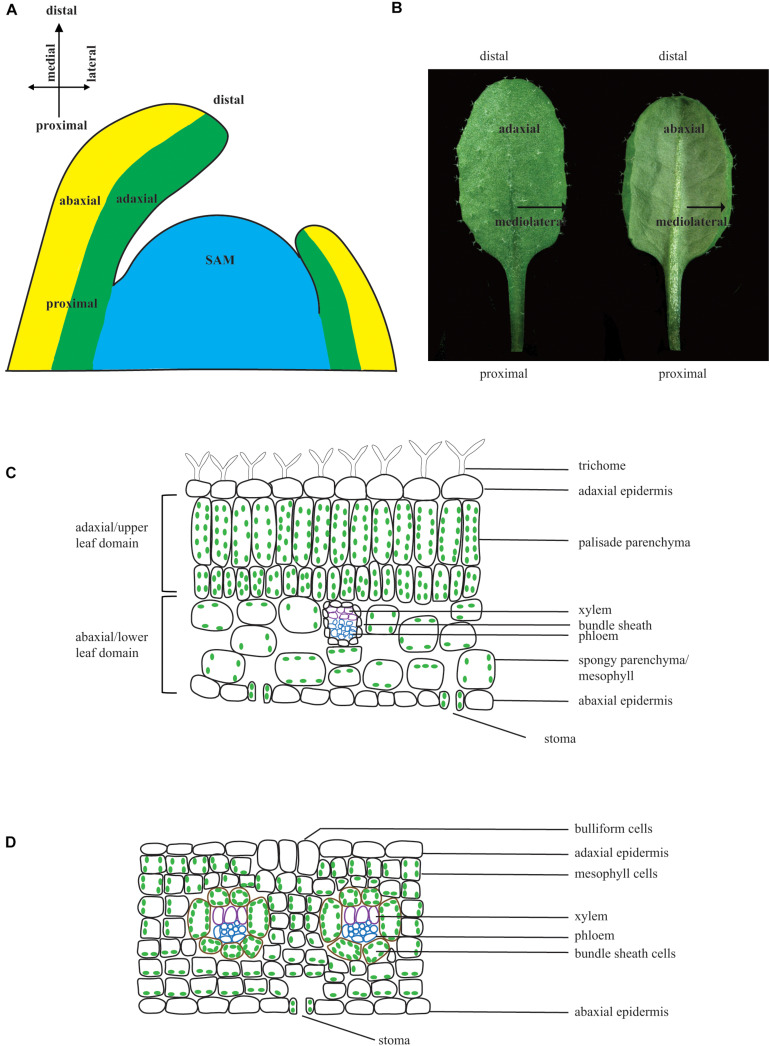
Leaf polarities. **(A)** Schematic diagram of a longitudinal section of a SAM showing the adaxial–abaxial domains, proximal–distal domains, and medial-lateral domains. **(B)** Arabidopsis leaf with adaxial side (left) and abaxial side (right). **(C)** Schematic diagram of cross section through a typical dicot C3 plant leaf. A leaf is enclosed by the upper (adaxial) epidermis and lower (abaxial) epidermis. The upper epidermis has trichomes. The upper mesophyll cells are tightly organized and enriched in chloroplast, whereas the lower mesophyll cells are loosely organized with fewer chloroplasts. Vascular bundles exhibit polarity with adaxially localized xylem and abaxially localized phloem. Stoma are abundant on the lower epidermis and trichome may or may not be present on the lower epidermis. **(D)** Schematic diagram of cross section through a monocot C4 plant leaf. Bulliform cells are present on the adaxial surface, and bundle sheath cells enclosing the vascular bundle are large and have chloroplasts.

Leaves are the major organ for photosynthesis and are specified for maximum capture of sunlight and minimum loss of water. Leaf morphology and development differ in the two main groups of angiosperms: parallel veined monocots and reticulate veined eudicots (Conklin et al., 2018). Proper leaf morphology is essential for light interception, transpiration rates, carbon fixation, photosynthesis, and crop yield and is established through numerous genetic pathways (Xu et al., 2018). In monocots, such as rice and maize, leaves form as strap-shaped structures with a sheathing leaf base around the shoot apical meristem (SAM), whereas eudicot leaves are initiated as bumps around the periphery of the SAM and ultimately form a flat structure composed of a lamina and petiole (Conklin et al., 2018; Xu et al., 2018). *Arabidopsis thaliana* (Arabidopsis) will be used here as representative of dicot leaf development (Christenhusz and Byng, 2016). Leaves are generated from a group of stem cells located at the tip of the shoot called the SAM. Approximately 500 cells constitute the Arabidopsis SAM and are divided into three functional and cytohistological zones: the central zone, the peripheral zone, and the rib zone (reviewed in Dodsworth, 2009; Barton, 2010; Du et al., 2018). The number of cells in the central zone is relatively constant, and cell division in this zone gives rise not only to stem cells that maintain the central zone but also to daughter cells that become incorporated in the peripheral zone. Cell division in the peripheral zone gives rise to lateral organs, including leaves and flowers. The rib zone cells are underneath the central zone and peripheral zones. After leaf primordia are initiated from the flanks of the peripheral zone, three polarities, the adaxial–abaxial polarity, the proximal–distal polarity, and the medial–lateral polarity, are established (Figure 1A). Compared to the deep understanding of the mechanisms of adaxial–abaxial polarity establishment, much less is known about how proximal–distal polarity and medial–lateral polarities are established. Several previous reviews have focused on adaxial–abaxial polarity (Byrne, 2006; Szakonyi et al., 2010; Liu et al., 2012; Du et al., 2018). Here, we focus on gene regulatory network regulating the establishment of all three axes during leaf development with an emphasis on recent advances in order to provide a basis for future studies.

## Establishment of Adaxial–Abaxial Polarity

**FIGURE 2:**
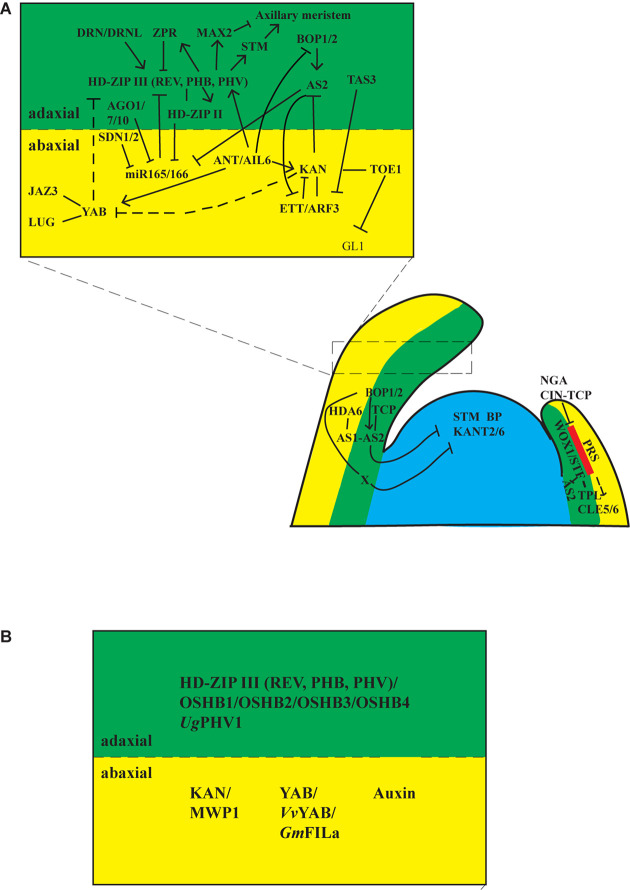
Regulatory networks for establishing leaf polarities. **(A)** Gene regulatory networks in Arabidopsis. Upper, Gene regulatory networks for adaxial/abaxial polarity. Adaxial identity is established by HD-ZIP III transcription factors, whereas the abaxial identity is established by KAN and YAB transcription factors. The activities of HD-ZIP III are repressed by *ZRP* proteins and miR165/166. HD-ZIP III proteins activate and interact with HD-ZIP II transcription factors to repress expression of miR165/166; thus, a feedback loop is formed between miR165/166-HD-ZIP III-HD-ZIP II. miR165/166 are processed and sequestered by AGO1, AGL7, AGO10, and other miRNA processing proteins. SDN1 and SDN2 proteins mediate the degradation of miR165/166. A second feedback loop functions between AS2-KAN-ETT. ETT is repressed by TAS3, ETT physically interacts with KAN to repress *AS2* and *KAN*, and AS2 represses *ETT*. AS2 also represses miR165/166. Thus, AS2 is a hub between these two feedback loops. KAN interacts directly with TOE1 to form a repressive loop at the *GL1* locus to suppress abaxial trichome formation in juvenile leaves. DRN and DRNL bind to HD-ZIPIII proteins and assist them in activating *STM* and *MAX2* to activate or suppress axillary meristem formation on the adaxial side of leaves. Lower, Gene regulatory networks for proximodistal and mediolateral polarities. Proximodistal polarity established by BOP1/2 and AS1/2 complexes. BOP1/2 proteins activate *AS2* directly, and AS2 physically interacts with AS1 and TCP transcription factors. AS1 associates with HDA6 to restrict class I *KNOX* genes (*STM*, *BP*, *KNAT2*, and *KNAT6*) in the SAM to suppress meristematic activities in leaves. BOP1/2 proteins function through AS2-dependent and AS2-independent pathways to repress class I *KNOX* genes. *WOX1* and *PRS* are expressed at the boundary between adaxial and abaxial domains. *WOX1* and *PRS* are restricted from the tip of the leaf by NGS and CIN-TCP transcription factors. TPL physically interacts with STF (WOX1) to repress *AS2* and promote lateral expansion of the lamina. CLE5 and CLE6 act downstream of WOX1 and PRS. Red area indicates the expression domain of *WOX1* and *PRS*. Filled lines represent direct interaction and dashed lines indicate indirect or genetic interaction. **(B)** Conserved gene regulatory network among plants. Adaxial polarity is established by HD-ZIP III (REV, PHB, PHV) transcription factors. Orthologs of the HD-ZIP III transcription factors, OSHB1/2/3/4 in rice, and UgPHV1 in *Utricularia* have similar roles as HD-ZIP III transcription factors in Arabidopsis. Abaxial polarity is established by YAB, KAN, and auxin pathways in Arabidopsis. The *KAN1* ortholog *MWP1* in maize, the *YAB* ortholog *VvYAB* in grapevine, and *GmFILa* in soybean have conserved roles as the YAB proteins in Arabidopsis. How auxin is involved in establishing adaxial–abaxial polarity is complicated. Nevertheless, it is required for polarity establishment in Arabidopsis and Chinese cabbage.

Adaxial–abaxial polarity is comparable to dorsiventral polarity in animals. When leaf primordia are initiated and separated from the SAM, the portion that is closest to the SAM is the adaxial domain, whereas the portion that is away from the SAM is the abaxial domain. Initially, leaf primordia fold over the SAM and later bend away from the SAM because of faster growth on the adaxial domain than the abaxial domain. As a result, the adaxial side becomes the upper side of the leaf, and the abaxial side becomes the lower side of the leaf (Figure 1B). As leaves are normally flattened during lamina outgrowth for maximum sunlight capture, the abaxial and adaxial side cells become morphologically different. In Arabidopsis leaves, cells on the adaxial (upper) domain are relatively elongated and tightly compacted, with many chloroplasts to optimize light capture and photosynthesis. Cells on the abaxial (bottom) domain are rounder and have large air spaces between them, with many stomata on this side to promote gas exchange (Figure 1C). Trichomes are always present on the adaxial surface of true leaves; however, they are not present on the abaxial surface until the plant is in the adult phase (Eshed et al., 2001; Kerstetter et al., 2001; Poethig, 2003; Liu et al., 2012). The leaf anatomy in the monocot C4 plant maize differs from that of Arabidopsis, as it uses different mechanisms for photosynthesis. Cells on the adaxial and abaxial domain are similar in size, and there are some bulliform cells on the adaxial surface, which may play a role in leaf rolling in response to water deficit stress (Xu et al., 2018). The bundle sheath cells in C3 plants are normally similar in size to the vascular cells and do not contain chloroplast, but the bundle sheath cells in C4 plants are larger than the vascular cells and normally contain chloroplast such that they can photosynthesize (Figure 1D). Although the morphological difference between adaxial and abaxial cells becomes apparent at later stages of leaf development, the expression of adaxial–abaxial identity genes is observed in globular-stage cotyledons and leaf primordia, suggesting that the establishment of adaxial–abaxial polarity occurs very early in leaf development (Eshed et al., 2001; Kerstetter et al., 2001; Emery et al., 2003). Molecular and genetic studies have shown that adaxial identity is established and maintained by a group of plant-specific homeodomain/leucine zipper (HD-ZIP) transcription factors, whereas abaxial identity is established and maintained by KANADI (KAN) family (GARP domain transcription factors) and YABBY (YAB) family transcription factors. Other factors are involved in the establishment of adaxial–abaxial identity by regulating or interacting with the above three groups of proteins (Figure 2A) (Talbert et al., 1995; Eshed et al., 2001, 2004; Kerstetter et al., 2001; McConnell et al., 2001; Emery et al., 2003; Prigge et al., 2005; Wenkel et al., 2007).

### Establishment of Adaxial Identity by HD-ZIP III and Related Factors

#### HD-ZIP III Transcription Factors Specify Adaxial Identity

The first protein that was reported to mediate adaxial–abaxial polarity was an MYB domain transcription factor PHANTASTICA (PHAN) in *Antirrhinum majus*. Mutations in *PHAN* converted flat leaves into needle-like structures (Waites and Hudson, 1995). In Arabidopsis, plant-specific class III HD-ZIP family proteins, including PHABULOSA (PHB)/ATHB14, PHAVOLULA (PHV)/ATHB9, REVOLUTA (REV)/ATHB8, and INCURVATA4/CORONA/ATHB15, establish adaxial identity (Emery et al., 2003; Prigge et al., 2005). Expression analysis showed that *PHB*, *PHV*, and *REV* are restricted to the adaxial domain of developing leaf primordia (Eshed et al., 2001; McConnell et al., 2001; Otsuga et al., 2001; Emery et al., 2003). Semidominant gain-of-function *phb*, *phv*, and *rev* mutants showed loss of abaxial identity and adaxialization of leaves (McConnell et al., 2001; Emery et al., 2003). Although recessive single loss-of-function mutants in these genes do not cause obvious defects in adaxial identity, the *phb phv rev* triple mutant exhibits severe phenotypes that include needle-like leaves without blades and absence of the SAM (Emery et al., 2003). Genetic studies showed that ATHB8 and ATHB15 act partially redundantly with PHB, PHV, and REV (Prigge et al., 2005). Specification of adaxial polarity is not only important for the generation of flat leaves for maximizing sunlight capture, but it is also important for the development of axillary meristems, which arise from the adaxial side of leaves. Consistent with this, axillary meristem activity was absent in the *phb phv rev* triple mutant (Emery et al., 2003; Prigge et al., 2005). The protein MORE AXILLARY BRANCHES2 (MAX2) is required for the production and perception of the strigolactone signal that suppresses axillary meristems. *REV* and *MAX2* were expressed in overlapping regions in leaf veins and petioles, and REV activates *MAX2* directly by binding to the *MAX2* promoter (Brandt et al., 2012; Hong et al., 2020). Consistent with MAX2 acting downstream of REV, radialization of vascular elements in *rev10D*-dominant mutants was suppressed by *max2* (Hong et al., 2020). In addition, REV directly activates *SHOOT MERISTEMLESS* (*STM*) in leaf axils to promote axillary meristem formation (Shi et al., 2016). This regulation appears to be promoted by DORNROSCHEN (DRN) and DORNROSCHEN-LIKE (DRNL), two AP2/ERF transcription factors that physically interact with REV and bind to a region near the transcriptional start site of *STM* (Zhang et al., 2018). The interaction of DRN/DRNL with REV promotes REV binding to *STM* regulatory regions (Shi et al., 2016; Zhang et al., 2018). The AP2 domain of DRN/DRNL interacts with the PAS domain of REV (Zhang et al., 2018). LITTLE ZIPPER3 (ZPR3) destabilizes the DRN/DRNL-REV interaction by competing for binding to REV (Zhang et al., 2018). Together, molecular and genetic studies have demonstrated that plant-specific class III HD-ZIP proteins establish adaxial identity and activate *STM* expression to promote axillary meristem formation (Figure 2A).

#### Regulation of HD-ZIP IIIs by miR165/166

Defects in adaxial/abaxial identity are also observed in plants lacking ARGONAUTE1 (AGO1), a key member of the AGO family that binds with regulatory small RNAs to form the RNA induced silencing complex (reviewed in Chen, 2009; Wang J. et al., 2019). Mutations in *AGO1* result in trumpet-like or radialized leaves, mimicking semidominant *phb-d* or *phv-d* leaves. miR165/166 are expressed in the abaxial domain of the leaf, complementary to that of *PHB* and *PHV*. Overexpression of a *PHB* construct containing sequence changes that reduce the complementary of the mRNA to miR165/166 but do not alter the amino acid sequence resulted in a *phb-d* phenotype (Mallory et al., 2004). Together, these lines of evidence indicate that PHB and PHV are directly regulated by miR165/166. miR165/166 also functions non-cell-autonomously to repress HD-ZIP III transcription factors. *MIR165A*, *MIR166A*, and *MIR166B* are expressed in the abaxial epidermis of leaves (Yao et al., 2009). Mature miR165 is detected in the abaxial epidermis and one to two cell layers underneath the epidermis (Kidner and Martienssen, 2004), suggesting that miR165 and possibly also miR166 migrate to neighboring cells and create an miR165/166 gradient. Consistent with this, confocal analysis on PHB-GFP protein showed that it is not evenly distributed in the adaxial domain of leaves (Miyashima et al., 2011; Tatematsu et al., 2015). A pair of artificially designed versions of miR165 and PHB were used to analyze their interaction. A mutated form of *PHB* (*PHBm*, which does not respond to endogenous miR165/166) was expressed in most of the leaf primordia. Introducing a mutated form of miR165 (miR165mu) complementary to *PHBm* gradually repressed the PHBm protein in the abaxial cells of leaves, indicating non-cell-autonomous function of endogenous miR165/166 (Miyashima et al., 2011; Tatematsu et al., 2015).

Proteins involved in transcriptional regulation of miR165/166, such as ASYMMETRIC LEAVES1 (AS1) and AS2, or in biogenesis of miR165/miR166, such as HASTY (HST), AGO1, AGO7, AGO10, and RNA-dependent RNA polymerase 6 (RDR 6), also function in modification of adaxial cell fate in leaves (Bollman et al., 2003; Li et al., 2005; Xu et al., 2006; Liu et al., 2009; Zhu et al., 2011). AGO10 is expressed at the adaxial side of leaf primordia and in the provasculature underneath the SAM to restrict the expression of miR165/166 to the abaxial side of leaves and to maintain stem cell fate in the SAM (Lynn et al., 1999). AGO10 has higher binding affinity to miR165/166 than AGO1 (Zhu et al., 2011), such that levels of miR165/166 are maintained to certain levels to restrict the expression of HD-ZIPIII to the adaxial side. The balance of miR165/166 is also maintained by the degradation activities of SMALL RNA DEGRADING NUCLEASE (SDN1) and SDN2 (Yu et al., 2017). AGO10 associated miR165/166 is more susceptible to SDN1 and SDN2 mediated trimming. The trimmed and unmethylated miR165/166 are then tailed by the nucleotidyl-transferase HESO1 (Yu et al., 2017). *AGO7* is also expressed on the adaxial side of leaf, and it acts synergistically with AS2 in promoting adaxial cell fate (Xu et al., 2006; Hoyer et al., 2019). Yeast-one-hybrid analysis showed that TEOSINTE BRANCHED/CYCLOIDEA/PCF (TCP) transcription factors bind to the “TGGTCC” motif of *AGO7*, and SQUAMOSA PROMOTER BINDING PROTEIN-LIKE (SPL) binds to the “GTCA” motif of *AGO7*. However, truncated *AGO7* promoter activity analysis showed that the SPL binding site is not required for the polar localization of *AGO7*. Additionally, the SPL and TCP binding sites are not strictly required for the function of AGO7 in leaves (Hoyer et al., 2019). It will be interesting to know what controls polar localization of AGO7 and AGO10 to understand how HD-ZIPIII transcription factors are regulated.

#### HD-ZIP III-ZPR Feedback Loop

The DNA-binding activities of HD-ZIP transcription factors require homodimerization through their leucine zipper domain (Tron et al., 2001, 2004, 2005). A group of LITTLE ZIPPER (ZPR) proteins ZPR1, ZPR2, ZPR3, and ZPR4 have a leucine zipper domain similar to that of HD-ZIP III. Transcripts of *ZPR* are localized to the adaxial portion of leaf primordia, like the *HD-ZIP III* transcription factors but at much lower levels. Plants ectopically expressing *ZPR3* had rod-like and downward curling leaves, indicating abaxialization. This could be explained by the activities of the ZPR proteins, in that the zipper domain of the ZPR proteins forms heterodimers with HD-ZIP III to prevent the homodimerization of HD-ZIP III proteins. Thus, the activities of HD-ZIP III proteins are reduced in *35S:ZPR3* (Wenkel et al., 2007; Kim et al., 2008; Magnani and Barton, 2011). *ZPR3* and *ZPR4* are induced by dexamethasone in the dexamethasone-inducible REV fusion plant, and *ZPRs* were also identified as direct targets of REV, thus forming a feedback loop that modulates adaxial identity protein activity (Figure 2A) (Wenkel et al., 2007; Kim et al., 2008).

#### HD-ZIP II Transcription Factors

Like class III *HD-ZIP* genes, class II *HD-ZIP* genes *HOMEOBOX ARABIDOPSIS THALIANA 2* (*HAT2)*, *HAT3*, and *ARABIDOPSIS THALIANA HOMEOBOX 4* (*ATHB4)* are expressed in the adaxial domain of leaf primordia (Brandt et al., 2012; Turchi et al., 2013). *HD-ZIP II* genes are regulated by a red/far-red light ratio that induces the shade avoidance response (Ruberti et al., 2012). These proteins are directly activated by REV and physically interact with REV to directly repress miR165/166, thus maintaining distinct adaxial and abaxial domains (Figure 2A) (Bou-Torrent et al., 2012; Brandt et al., 2012; Turchi et al., 2013; Merelo et al., 2016).

### Establishment of Abaxial Identity by KANADI and YABBY Proteins

#### KANADI Proteins

The flattened leaf lamina results from the parallel development of adaxial and abaxial cells. Abaxial identity is established by members of two gene families, the *KANADI*s and the *YABBY*s (Eshed et al., 2001, 2004; Emery et al., 2003; Kerstetter et al., 2001; Prigge et al., 2005; Izhaki and Bowman, 2007). The KAN family shares a common GARP domain of MYB transcription factors, and three members of this family, *KAN1*, *KAN2*, and *KAN3*, are expressed in the abaxial portion of leaf primordia (Kerstetter et al., 2001; Eshed et al., 2004). None of the *kan1*, *kan2*, *kan3*, and *kan4* single mutants exhibited a dramatic loss of abaxial polarity phenotype; however, the leaves of the *kan1/2* double mutant are narrow and have ectopic outgrowths on their abaxial side. The *kan1/2/3* triple mutant looked like the *kan1/2* double mutant, whereas *kan1/2/4* and *kan1/2/3/4* mutants produced radialized rosette leaves or radialized leaf-like structures on the hypocotyl (Eshed et al., 2001, 2004; Izhaki and Bowman, 2007). Overexpression of *KAN1*, *KAN2*, or *KAN3* resulted in radialized leaves without blades, and the plants lacked an SAM (Eshed et al., 2001, 2004; Emery et al., 2003; Kerstetter et al., 2001; Izhaki and Bowman, 2007). Thus, KAN1, KAN2, KAN3, and KAN4 function redundantly in specifying leaf abaxial identity.

*KAN1* is an abaxial identity gene, but the *kan1* mutant was first identified in screens for juvenile-to-adult phase change mutants (Kerstetter et al., 2001). Trichomes are polarly located on the adaxial side of juvenile leaves, whereas trichomes are present on both sides of adult leaves. The production of trichomes on the abaxial side of leaves is a hallmark for adult leaves and is promoted by a group of miR156-targeted SPL transcription factors (Wu et al., 2009). SPL9 and SPL15 directly activate the expression of *miR172b*, a precursor for mature miR172 (Wu et al., 2009; Hyun et al., 2016). Mutations in miR172-targeted *AP2* genes, such as *target of eat1* (*toe1*) and *toe2*, slightly accelerate abaxial trichome production (Wu et al., 2009), whereas the *toe1 toe2*, *toe1 toe2 ap2*, and the *toes toe2 smz snz* mutants are gradually more accelerated in abaxial trichome production (Wang L. et al., 2019). Abaxial trichome production is accelerated in *kan1* (Kerstetter et al., 2001; Wang L. et al., 2019), and KAN1 is found to associate with TOE1 in yeast two-hybrid screens. The N-terminal domain of KAN1/2 binds to miR172-targeted AP2 proteins, whereas KAN3/4 do not bind to them. ChIP and EMSA assays showed that both KAN1 and TOE1 bind to the 3′ end of a trichome initiation gene *GLABRA1* (*GL1*), and mutations in this KAN1/TOE1-binding site resulted in precocious abaxial trichome production (Wang L. et al., 2019; Xu et al., 2019). Since KAN1 and TOE1 bind to the 3′ end of *GL1*, how do they repress the transcription of *GL1*? A chromosome conformation capture (3C) assay showed that the 3′ end of *GL1* was brought to its 5′ end by the KAN1–TOE1 complex. In a 3C assay, chromatin is crossed linked, digested with restriction enzymes, and ligated. The ligation product is then subjected to quantitative polymerase chain reactions by primers spanning the 5′ end and 3′ end to give 100- to 150-bp amplicons. Truncation of the 3′ end significantly reduced the amount of 5′–3′ amplicons, whereas insertion of an 8-kb fragment between the 5′ end and 3′ end did not interfere with the activity of KAN1–TOE1 complex, indicating that *cis*-elements at the 3′ end are required for the formation of the KAN1–TOE1 repressing loop and *cis*-elements in the middle are not critical. Therefore, the KAN1–TOE1 complex is involved with the temporal and spatial production of abaxial trichomes. In juvenile leaves where miR156 levels are high, levels of TOE1 are also high, and TOE1 directly interacts with KAN1 on the abaxial side of the leaf to form a repressive loop at *GLABRA1* (*GL1*). In adult leaves, when levels of miR156 are low, TOE1 activity is also low, which deactivates the KAN1–TOE1 repressive loop and derepresses *GL1* (Figure 2A) (Wang L. et al., 2019; Xu et al., 2019).

#### YABBY Proteins

The *YABBY* gene family encodes HMG-like proteins, and members of this family in Arabidopsis are initially expressed in the whole leaf primordium and later restricted to the abaxial domain as the primordium elongates (Sawa et al., 1999; Siegfried et al., 1999). The *fil*/*yab1*, *yab2*, *yab3*, and *yab4* single mutants do not have visible defects in leaf polarity; the *yab1/3* double mutant, however, exhibits some loss of abaxial cell identity, while the lamina looks normal compared to wild type. The *yab1/2/3/5* quadruple mutant shows more severe defects with fully radialized leaves, indicating functional redundancy among members of this gene family (Siegfried et al., 1999; Sarojam et al., 2010). The failure of leaf laminar outgrowth in loss-of-function *yab* mutants (*yab1/2/3/5* and *pANT* > > *miR-YAB1*) is associated with loss of leaf margin cells and discontinuous vein patterning, which might be caused by lower auxin levels in their leaves (Goldshmidt et al., 2008; Sarojam et al., 2010). *WUSCHEL* (*WUS*), a gene required for SAM maintenance, is restricted to the SAM in WT but reactivated in *pANT* > > *miRYAB1* leaf primordia, suggesting that YAB proteins suppress meristematic activity in leaf primordia to promote proper leaf development (Sarojam et al., 2010). Ectopic expression of *YAB1* or *YAB3* under the *35S* promoter resulted in the abaxialization of leaves and loss of the SAM (Siegfried et al., 1999). Interestingly, the *yab1/3/5* triple mutation suppresses the abaxial outgrowths seen on *kan1/2/4* leaves. The leaves of the *kan1/2/4 yab1/3/5* sextuple mutant are rod-like (Izhaki and Bowman, 2007), indicating a synergistic interaction between YAB and KAN families in establishing abaxial cell fate. Additionally, mutations in the auxin biosynthetic *YUCCA* (*YUC*) genes can also suppress the outgrowths in the *kan1/2* double mutant, and the *yuc1/2/4* triple mutation enhances the loss of abaxial identity in a *kan1/2* background (Wang et al., 2011).

It is not known if the synergistic interaction between YAB family proteins and KAN family proteins is attributable to a physical YAB–KAN interaction. *FIL/YAB1* is ectopically expressed in *kan1/2* leaf outgrowths, suggesting that *YAB* might be repressed by KAN (Eshed et al., 2004). YAB proteins physically interact with the GRO-TUP1–like proteins LEUNIG (LUG) and LEUNIG-HOMOLOG (LUH) to repress *PHB* (Stahle et al., 2009). On the other hand, the activities of YAB proteins are attenuated by JASMONATE-ZIM DOMAIN protein JAZ3 (Boter et al., 2015). *AINTEGUMENTA* (*ANT*), which encodes an APETALA2/ETHYLENE RESPONSE FACTOR transcription factor, functions redundantly with three other *AINTEGUMENTA-LIKE* (*AIL*) family members, *AIL5*, *AIL6*, and *AIL7*, in floral organogenesis and leaf development (Krizek, 2009, 2015; Mudunkothge and Krizek, 2012). *ANT*, *AIL5*, and *AIL6* were initially expressed throughout incipient leaf primordia (Long and Barton, 2000; Prasad et al., 2011). The *ant ail6 ail7* triple mutant produces fewer rosette leaves, which are narrower than those in *ant* single mutants or *ant ail6* double mutants (Mudunkothge and Krizek, 2012; Krizek, 2015). An *ant* mutation enhances defects in leaf polarity in *fil* and *fil yab3* mutant backgrounds through synergistic regulation of *PHB* (Nole-Wilson and Krizek, 2006). RNA-seq analysis in whole inflorescence and ChIP-Seq analysis in stage 6/7 flowers revealed that ANT binds directly to the adaxial polarity genes *PHB* and *BLADE-ON-PETIOLE1* (*BOP1*) and to the abaxial polarity genes *KAN2* and *YAB3* (Figure 2A) (Krizek et al., 2020), suggesting that *ANT* is involved in regulating organ polarity. Together, the studies of ANT and AIL proteins in Arabidopsis suggest that they have a role in establishing adaxial/abaxial polarity in lateral organs, but how they are involved in this process remains to be resolved.

### AS2 Functions as a Hub for Juxtaposition of Adaxial–Abaxial Cell Fate

The adaxial and abaxial cell fates are specified by the interaction of distinct transcription factor families, with an LOB domain transcription factor ASYMMETRIC LEAVES2 (AS2) seeming to function as a hub (Figure 2A). *AS2* is expressed in the adaxial domain of leaves (Iwakawa et al., 2007; Wu et al., 2008; Jun et al., 2010; Husbands et al., 2015) and directly represses miR166 to restrict expression of miR166 to the abaxial domain, resulting in the expression of *HD ZIP III* genes on the adaxial domain (Eshed et al., 2001; McConnell et al., 2001; Otsuga et al., 2001; Emery et al., 2003; Wu et al., 2008; Jun et al., 2010; Husbands et al., 2015). *KAN1*, which is expressed on the abaxial domain, represses *AS2* transcription directly to restrict its expression to the adaxial domain (Wu et al., 2008). AS2 also functions synergistically with RDR6 in adaxial patterning (Li et al., 2005). RDR6 is involved in *trans-*acting siRNA (TAS1–TAS4) biogenesis (Yoshikawa et al., 2005; Chen, 2009). *TAS3* is expressed in the adaxial domain of leaves and represses the expression of the *AUXIN RESPONSE FACTORS* (*ARFs*) *ETTIN* (*ETT*, also termed *ARF3*) and *ARF4* (Fahlgren et al., 2006; Garcia et al., 2006; Hunter et al., 2006). *ARF3* is expressed in the abaxial domain and physically interacts with KAN proteins to facilitate KAN activity (Pekker et al., 2005; Garcia et al., 2006; Kelley et al., 2012). AS2 represses *ETT* directly, and AS2 activity is required for the DNA methylation of *ETT* on its sixth exon (Iwasaki et al., 2013). Two nucleolus localized proteins, NUCLEOLINI and RNA HELICASE10 are associated with the AS2-dependent DNA methylation of ETT (Vial-Pradel et al., 2018). It is also reported that replication factor C subunit 3 and DNA polymerase subunit INCURVATA2 function as modifiers of AS2 in repressing ETT and ARF4 (Luong et al., 2018). Therefore, a feedback loop among ETT-KAN-AS2 acts to spatially define adaxial–abaxial cell fates.

Other factors, such as AS1, TCP, and BLADE-ON-PETIOLE 1 and 2 (BOP1/2) transcription factors, and histone acetylase/deacetylase epigenetic factors also interact with AS2 directly or indirectly to specify adaxial–abaxial polarity. AS1 (the ortholog of PHAN in Arabidopsis) and miR319-targted TCP transcription factors physically interact with AS2 in adaxial–abaxial patterning (Xu et al., 2003; Guo et al., 2008; Li et al., 2012). The BTB/POZ domain protein BOP1, however, binds to the AS2 promoter directly to activate *AS2* expression (Ha et al., 2007; Jun et al., 2010). At least three lines of evidence indicated that AS1/AS2 interacts with epigenetic factors in establishing adaxial cell fate. First, treating an *as2* mutant with trichostatin A (a histone deacetylase inhibitor) resulted in radialized leaves, indicating enhanced loss of adaxial polarity (Ueno et al., 2007). Second, AS1 interacted with histone deacetylase HDA6 *in vitro* and *in vivo*, and the AS1–HDA complex was recruited to repress the *KNOX I* genes in leaf patterning (Luo et al., 2012). Third, a histone acetylase ELONGATE was reported to function redundantly with AS1 and AS2 in establishing adaxial cell fate (Kojima et al., 2011).

Mutant screens in *as2* backgrounds led to the discovery of ribosomal proteins (r-proteins), whose mutation enhances leaf abaxial polarity in *as1* or *as2* backgrounds (Horiguchi et al., 2011). AS2 is localized to the nucleus and forms AS2 bodies around the peripheral region of the nucleolus. Truncation analysis on AS2 motifs revealed that ICG and LZL regions are required for the nuclear localization of AS2. The zinc-finger DNA-binding motif of AS2 is strongly conserved among AS2/LOB protein family, and the sequences within and close to this motif are essential for the formation of AS2 bodies, which in turn is essential for the function of AS2 (Luo et al., 2020). Florescence *in situ* hybridization analysis showed that AS2 bodies are close to 45S rDNA, and they both are close to chromocenters. Therefore, the synergistic interaction between AS2 and ribosomal proteins might be caused by overlapping actions of AS2 bodies and ribosomal DNA in the chromocenter, which might be important for the epigenetic repression of its targets (Luo et al., 2020). Other two ribosome-related mutants, *oligocellula2* (*oli2*) and *g-patch domain protein* (*gdp1*), were also found in the sensitized *as2* mutant screens. OLI2 and GDP1 function synergistically to promote cell proliferation, and mutations in them caused narrow leaves. However, they do not strongly enhance the polarity defect of *as2* (Kojima et al., 2018), indicating that leaf polarity induced by r-proteins function in a different pathway to OLI2 and GDP1.

### Role of Auxin in Specifying Adaxial–Abaxial Identity

The plant hormone auxin is required for lateral organ initiation and many other aspects of plant development (Reinhardt et al., 2000; Benkova et al., 2003; Friml et al., 2003; Simonini et al., 2016, 2017, 2018). Several lines of evidence suggest that auxin is also involved in leaf patterning along the adaxial–abaxial axis. It was reported in 2007 that *PIN-FORMED1* (*PIN1*), an auxin efflux carrier, and the auxin reporter *DR5* are abnormally localized in *kan1/2/4* and *rev phb phv* triple mutants (Izhaki and Bowman, 2007). DR5 and PIN1 were also abnormally distributed in the *yab1/2/3/5* quadruple mutant in several organs, such as embryo, leaf primordium, and leaf vein, consistent with their roles in cotyledon development, leaf adaxial–abaxial differentiation, and leaf margin patterning (Sarojam et al., 2010). Auxin seems localized on the adaxial domain of leaf primordia as indicated by the distribution of DII (an auxin sensor) and PIN1 (Qi et al., 2014; Shi et al., 2017). However, other studies argue that auxin is not polarly localized because maximum auxin levels are found between the REV and KAN1 domains, and quantitative analysis on the auxin sensor R2D2 showed that there is no polar localization of R2D2 in lateral organ primordia, although there is transient asymmetric distribution of mDII (Caggiano et al., 2017; Bhatia et al., 2019). How auxin could be involved in lateral organ development (including leaf development) has been discussed by Heisler and Byrne (2020), and whether auxin is asymmetrically distributed in leaf primordia remains to be resolved. REV directly activates two auxin biosynthesis genes, *TAA1* and *YUC5* (Brandt et al., 2012). KAN1, a transcriptional repressor, regulates biosynthesis, transport, and signaling of auxin. The auxin response factor ARF3/ETT physically interacts with KAN proteins on the abaxial domain of leaves (Kelley et al., 2012; Huang et al., 2014). RNA-seq analysis of cells sorted by different fluorescence markers (*pREV*:*REV-2YPET* and *pKAN1*:KAN1–*2GFP*) revealed that auxin-related genes are regulated by both REV and KAN1. REV activates auxin-related genes initially but represses them at a later time point. KAN1 functions antagonistically to REV in regulating a common group of targets (Ram et al., 2020). Auxin acts through binding to TIR1/AFB F-box proteins, leading to the release of Aux/IAA from ARFs. There are 23 ARFs in the Arabidopsis genome. Among them, ARF3, ARF4, and ARF5 seem to have roles in adaxial–abaxial patterning (Pekker et al., 2005; Garcia et al., 2006; Guan et al., 2017). ARF3/ETT is atypical as it does not interact with Aux/IAA (Simonini et al., 2016). Sequence analysis of ETT combined with yeast two hybrid assays showed that a 27-kDa ETT variant ETT_388-594, encompassing the entire predicted ETT-specific domain, contains auxin responsive elements (Simonini et al., 2018). RNA-seq analysis on *ett* mutants and ChIP-seq analysis on ETT-GFP transgenic plants revealed that ETT regulates a wide range of genes, which include ethylene, cytokinin, and auxin biosynthesis, response, and signaling genes. ETT also binds to a bHLH proteins that interact with ETT. Among the polarity genes, *KAN3* is a direct target of ETT and is upregulated in *ett* (Simonini et al., 2017). KAN proteins also interact with ETT physically, and their interactions have a role in ovule development (Kelley et al., 2012). The RNA-seq, ChIP-seq, and genetic interactions between ETT and KAN were analyzed in reproductive tissues; therefore, mechanisms of how auxin is involved in adaxial–abaxial cell fate determination in leaves remain to be investigated by molecular and genetic studies.

### Adaxial–Abaxial Polarity in Plants Other Than Arabidopsis

Studies in species other than Arabidopsis showed that the mechanisms for establishment and maintenance of adaxial–abaxial polarity are conserved among some plants. The HD-ZIP III transcription factors in rice are encoded by *OSHB* genes. Similar to *HD-ZIP III* genes in Arabidopsis, *OSHB1/OSHB2/OSHB3/OSHB4* are expressed in the adaxial domain of rice leaves (Itoh et al., 2008). Ectopic expression of these *OSHBm* genes resulted in rod-like leaf structures, similar to the semidominant *rev*, *phv*, and *phb* mutations in Arabidopsis (Itoh et al., 2008). A calpain-like cysteine protease ADAXIALIZED LEAF 1 (ADL1) is required for the maintenance rather than establishment of adaxial polarity as the expression of *OSHB* is increased in mature *adl1* leaves, but not in immature *adl1* leaves. As a result, the *adl1* leaf is adaxialized (Hibara et al., 2009). Rolling of leaves in rice indicates loss of adaxial–abaxial identity. Several proteins in rice, including HD-ZIP IV family member Roc8, a KAN ortholog SHALLOT-LIKE1 (SLL1), and a CHD3/Mi-2 chromatin remodeling factor ROLLED FINE STRIPED (RFS), have been discovered to play essential roles in controlling rolling of leaves in rice (Zhang et al., 2009; Cho et al., 2018; Sun et al., 2020). The *milkweed pod1* (*mwp1*) gene encodes a KAN ortholog in maize. Molecular and genetic analysis of *mwp1* showed that it is required for maintenance of the abaxial identity of the maize leaf (Candela et al., 2008). Evolutionary studies on *KAN* family genes in ferns and lycophytes revealed that *KAN* family genes are expressed in the abaxial domain in ferns but not in lycophytes, suggesting that *KAN* family genes are conserved within ferns and some angiosperms, but not in lycophytes (Zumajo-Cardona et al., 2019). Similar results were also observed for the *YAB* gene family in that the grapevine *VvYABBY* gene and the soybean *YAB* family gene *GmFILa* are expressed in the abaxial domain of leaves, and overexpression of those genes in tomato or Arabidopsis resulted in abaxialization of leaves (Xiang et al., 2013; Yang et al., 2019; Zhang S. et al., 2019). Together, these studies suggest conserved roles of *KAN* and *YAB* genes in some angiosperms (Figure 2B).

Chinese cabbage (*Brassica rapa* ssp. *pekinensis*) produces flattened leaves early in the rosette stage, and the leaves become curved inward and folded at the heading stage, indicating adaxialization of leaves. External application of auxin to Chinese cabbage and RNA-seq analysis on the heading mutant *flat growth-1* (*fg-1*) revealed that auxin plays an essential role in establishing adaxial–abaxial polarity (heading) in Chinese cabbage (He et al., 2000; Li et al., 2019), indicating a conserved role of auxin in establishing adaxial–abaxial polarity. Although the molecular nature of *FG* is still unknown, six adaxial identity genes *BrREV*, *BrHB8.1*, *BrHB8.2*, *BrHB9*, *BrHB14.1*, and *BrHB14.2* and six abaxial identity genes *BrKAN1*, *BrBOP2*, *BrYAB1.1*, *BrYAB1.2*, *BrYAB2*, and *BrYAB3* were differently expressed in *fg-1* mutants, suggesting that heading of Chinese cabbage is caused by ectopic expression of adaxial/abaxial polarity genes.

*Utricularia gibba* produces both planar and non-planar (needle-like or cup-shaped) leaves. Planar leaves are formed when the expression of *UgPHV1* is restricted at the adaxial domain, and needle-like leaves are formed when *UgPHV1* is ectopically expressed, similar to the role of PHV in Arabidopsis (Whitewoods et al., 2020). Computational modeling on *U. gibba* planar leaves and non-planar leaves, together with molecular and genetic studies, revealed that *UgPHV1* patterns the leaf via regulating growth rate along orthoplanar (adaxial–abaxial), proximodistal, and perpendicular to both (mediolateral) axes. Planar leaves are formed when growth rates along the orthoplanar and perpendicular axes are higher than the growth rate along the proximodistal axis. Non-planar leaves are formed when the growth rate along the orthoplanar axis is higher than the growth rates along the proximodistal and perpendicular axes (Whitewoods et al., 2020). These results suggest that adaxial–abaxial polarity is formed by restricting growth rate along the proximodistal axis and that the adaxial identity gene *PHV1* has a role in restricting growth along the proximodistal axis. This work indicates that polarities are the results of oriented growth, rather than the cause.

## Establishment of Proximal-Distal Polarity

Proximal–distal polarity is established as the primordium grows away from the SAM. The domain close to the SAM is the proximal part, which gives rise to the petiole. The domain farthest from the SAM is the distal part, which gives rise to the blade. The best-known genes that control proximal–distal polarity are *BOP1/2*, as the *bop1 bop2* double mutant exhibits blade growth on the petiole, diminishing the petiole domain (proximal domain). *BOP1/2* genes are expressed at organ boundaries and have a wide range of roles in Arabidopsis development, including leaf patterning, floral organ patterning, floral meristem identity, floral organ abscission, and pedicel patterning (Hepworth et al., 2005; Norberg et al., 2005; Ha et al., 2007; Jun et al., 2010). *BOP1/2* genes are expressed at the proximal and adaxial side of leaf primordia, and their activities in the adaxial domain activate the expression of another adaxial gene *AS2* (Ha et al., 2007; Jun et al., 2010). *AS2* is expressed throughout initiating leaf primordia but becomes localized to the adaxial L1 layer in older leaf primordia (Iwakawa et al., 2007; Wu et al., 2008; Jun et al., 2010). Mutations in *AS2* resulted in an upward curling petiole and sometimes a small leaflet on the petiole (Lin et al., 2003; Xu et al., 2003; Iwakawa et al., 2007). Both the *bop1 bop2* and *as2* leaf phenotypes are associated with ectopic expression of meristem class I homeobox *KNOX* genes, including *SHOOT MERISTEMLESS (STM), BREVIPEDICELLUS (BP)*, *KNAT2*, and *KNAT6* (Semiarti et al., 2001; Byrne et al., 2002; Iwakawa et al., 2007; Ikezaki et al., 2010; Jun et al., 2010). The AS1–AS2 complex directly represses class I *KNOX* genes to suppress ectopic growth on the petiole (Guo et al., 2008). This is supported by genetic analysis showing that mutations in class I *KNOX* genes suppress the ectopic growth on *bop1 bop2* and *as2* petioles (Ha et al., 2010; Ikezaki et al., 2010). BOP1/2 proteins not only directly activate AS2 but also function redundantly with AS1 and AS2 to pattern the proximodistal axis, as growth on *bop1 bop2 as1* and *bop1 bop2 as2* petioles is much more dramatic than on the *bop1 bop2* double mutant or on *as1* or *as2* single mutants (Figure 2A) (Ha et al., 2007).

Leaves of maize and rice do not have petioles. Instead, they have a sheath that wraps around the shoot in place of a petiole. Loss-of-function studies in *OsBOP1/2/3* mutants revealed that these genes promote the growth of the distal blade. Leaf 1 of wild-type cultivated rice has only a sheath, whereas leaf 1 of the *osbop* triple mutant produces blades without a sheath. Overexpression of *OsBOP1* in rice does not affect the growth of the sheath; however, the length of the blade is shorter (Toriba et al., 2019). Thus, the sheath to blade ratio is affected by loss-of-function and gain-of-function mutations in *OsBOP*. Rhizomes are modified stems (without any blade) growing underground. Rhizomes of *Oryza longistaminata* have much higher levels of BOP1 than the leaves above ground, suggesting that BOP1 suppresses blade growth in rhizomes (Toriba et al., 2020). The growth of the sheath is also correlated with high levels of miR156, and OsSPL14 represses the expression of *BOP1/2/3* genes (Toriba et al., 2019), suggesting the role of miR156-SPL in establishing the proximal–distal polarity.

## Establishment of Medial-Lateral Polarity

The blade of a leaf expands laterally to increase its surface area for maximum capacity of photosynthesis. Two *WUSCHEL-RELATED HOMEOBOX (WOX)* genes, *WOX1* and *PRESSED FLOWER* (*PRS*) (also known as *WOX3*), were found to be involved in establishing mediolateral polarity. *PRS* encodes a homeodomain protein that shares 68% identity with WUS. *WOX1*, *PRS*, and the *PRS* ortholog *MAEWEST (MAW)* in Petunia are expressed at the boundary between adaxial–abaxial domains at the P2 stage and at the leaf margin at later stages. The *wox1 prs* double mutant and *maw* single mutant have narrower lamina than wild type, as well as an abnormal abaxial cell fate in lamina (Matsumoto and Okada, 2001; Vandenbussche et al., 2009; Nakata et al., 2012; Caggiano et al., 2017). Domain localization of expression of the maize orthologs of *PRS*, *NARROW SHEATH1* (*NS1*) and *NS2* is similar to that of *PRS*, and leaves of a *ns1 ns2* double mutant are much narrower than wild type (Nardmann et al., 2004). The *WOX1* orthologs in tobacco (*LAMINA1*), petunia (*MAEWEST*), *Medicago truncatula* (*STENOFOLIA, STF*), and barley (*NARROW LEAFED DWARF1*) have conserved roles in promoting lamina expansion along the mediolateral axis (Vandenbussche et al., 2009; Tadege et al., 2011a,b; Yoshikawa et al., 2016). In *M. truncatula*, STF recruits TOPLESS (TPL) and requires the activity of TPL to promote cell proliferation at the adaxial–abaxial boundary (Zhang et al., 2014). ANGUSTIFOLIA3 (AN3) physically interacts with LEUNIG (LUG), functioning redundantly with WOX1 and PRS to promote lamina lateral growth (Zhang F. et al., 2019). Two *CLAVATA3/ESR-RELATED* peptide ligand gene family members, *CLE5* and *CLE6*, were activated by WOX1 and PRS. However, the *cle5* and *cle6* single or double mutants did not show any defect in leaf development, probably due to the redundancy within the *CLE* family (DiGennaro et al., 2018). In most plants, the lamina margin is determinant and will not give rise to any new structures. The meristem activity in leaves is restricted by several NGATHA (NGA) and CINCINNATA-class-TCP (CIN-TCP) transcription factors, which act redundantly to restrict the activities of PRS in the leaf margin (Alvarez et al., 2016). Together, studies in several species suggest that leaf development along the mediolateral axis is promoted by PRS and WOX1. NGA and CIN-TCP transcription factors restrict the expression of *PRS*, whereas CLE5 and CLE6 are activated by PRS and WOX1. TPL, AN3, and LUG act redundantly with PRS and WOX1 to restrict the expression of *AS2* at lateral ends to promote the lateral growth of lamina (Figure 2A).

## Future Perspectives

Genetic studies in Arabidopsis showed that plants that cannot specify an adaxial fate, such as the *phb phv rev* triple mutant and *35S:KAN1* ectopic expression construct, or plants with loss of both adaxial and abaxial identities, such as the *kan1/kan2/kan4/phb/phv/rev* sextuple mutant, do not produce SAMs, suggesting that specification of polarity might be a prerequisite for SAM formation (Talbert et al., 1995; Kerstetter et al., 2001; Otsuga et al., 2001; Emery et al., 2003). However, none of these polarity genes are expressed in the central zone of the SAM, which is essential for maintenance of the SAM. How these adaxial–abaxial identity genes affect SAM formation is uncertain. One hypothesis is that a mobile signal moves from leaf primordia to the SAM and regulates the transcription or activity of meristematic genes. *WUS* is expressed in a small group of central cells in and underneath the L3 layer of the SAM, and it is required for maintenance of the SAM (Mayer et al., 1998). Although YAB1 does not move, the expression of *WUS* is expanded in *yab1/3* double mutants, indicating that downstream targets of YAB might be mobile and mediate the change of *WUS* expression (Goldshmidt et al., 2008). But what these downstream mobile targets are and how they maintain the SAM remain unknown. In 1954, Sussex proposed that signals from the SAM determine adaxial–abaxial polarity, suggesting a feedback loop between SAM identity genes and leaf adaxial–abaxial identity genes. Auxin and a lipophilic signal were proposed to be candidates for the “Sussex signal,” but it remains to be determined (Kuhlemeier and Timmermans, 2016). Moreover, the establishment of adaxial–abaxial polarity, proximodistal polarity, and mediolateral polarity seems to be coordinated rather than independent of each other. BOP1/2 are involved in both adaxial–abaxial polarity and proximal–distal polarity, and the leaves of a *phb phv rev* triple mutant are rod-like without blade outgrowth, indicating the loss of adaxial polarity, distal polarity, and lateral polarity at the same time. Ectopic expression of *ARF5/MP* resulted in loss of normal adaxial–abaxial and mediolateral development at the same time (Guan et al., 2017; Shi et al., 2017). Mechanisms of how these three polarities (adaxial–abaxial, proximal–distal, and mediolateral) are coestablished remain to be discovered. Computational modeling on *U. gibba* indicated that leaf shape variation (sheet-like or needle like) is correlated with different growth rates along the growth axes. A combination of computational modeling with molecular and genetic approaches could enable us to uncover how polarity regulators promote or restrict growth rates in certain areas that eventually shape leaves along the polarity axes. Studies on the development of unifacial leaves suggest strikingly different mechanisms in leaf patterning. In bifacial leaves, loss of adaxial identity resulted in rod-like leaves, whereas in unifacial leaves, loss of adaxial identity resulted in either cylindrical leaves (*Juncus wallichianus*) or flattened leaves (*Juncus prismatocarpus*). Molecular studies showed that *DROOPING LEAF* (*DL*), a *YABBY* paralog, is expressed in the middle of flattened leaves (*J. prismatocarpus*), like the expression of *PRS* in Arabidopsis. However, a similar expression pattern of *DL* was not observed in cylindrical leaves (*J. wallichianus*). Consistently, *PRS* is detected in the flattened leaf margin while not in the cylindrical leaf margin (Yamaguchi et al., 2010). Further analysis on cell division direction revealed that there is more thickening cell division on the adaxial domain than on the abaxial domain, which gives rise to flattened leaves like bifacial leaves (Yin and Tsukaya, 2019). Further investigation on unifacial leaf development may provide new insight into how leaves are flattened. Compared to the mechanisms in adaxial–abaxial polarity, much less is known about leaf development along the proximodistal polarity and/or mediolateral polarity axes, due to the lack of mutants. Other strategies, such as conditional screening of mutants combined with RNA-seq or studying plant species that have natural variation in proximodistal polarity and/or mediolateral polarity, may lead us to uncover new or redundant genetic pathways that pattern the leaf along the three polarity axes. Leaf shape diversity sometimes is associated with developmental phase transitions and stress responses (Wang et al., 2009; Wu et al., 2009; Kumar and Wigge, 2010; Xu et al., 2016; Leichty and Poethig, 2019). Understanding the mechanisms in leaf shape patterning may provide vital insight into developmental phase transitions and environmental plasticity.

## Author Contributions

MX wrote the first draft of the manuscript and drew the figures. Both authors revised the manuscript.

## Conflict of Interest

The authors declare that the research was conducted in the absence of any commercial or financial relationships that could be construed as a potential conflict of interest.
